# Recent advances in copper-catalyzed C–H bond amidation

**DOI:** 10.3762/bjoc.11.240

**Published:** 2015-11-17

**Authors:** Jie-Ping Wan, Yanfeng Jing

**Affiliations:** 1 College of Chemistry and Chemical Engineering, Jiangxi Normal University, Nanchang 330022, P.R. China

**Keywords:** amidation, C–H bond, cascade reactions, Copper catalysis, intermolecular, intramolecular

## Abstract

Copper catalysis has been known as a powerful tool for its ubiquitous application in organic synthesis. One of the fundamental utilities of copper catalysis is in the C–N bond formation by using carbon sources and nitrogen functional groups such as amides. In this review, the recent progress in the amidation reactions employing copper-catalyzed C–H amidation is summarized.

## Introduction

The amide functional group is a fundamental fragment in nature and in both living systems and artificial chemicals. Owing to their naturally relevance to biological processes, the amides have attracted extensive research interest in numerous areas such as chemical, pharmaceutical, biological and material sciences [1–5]. For the sake of synthesizing functional amides, tremendous efforts have been made by chemists who developed many distinct methodologies towards these compounds. Typical examples on amide synthesis are the direct amidation of acids/esters/acyl chloride/anhydrides [6–11], nitrile hydrolysis [12–16], Goldberg C–N cross coupling reaction [17], aldehyde/ketone amidation [18–23], the transamidation [24–29], and oxime rearrangement [30–33], to name only a few. It is obvious that the known strategies in amide synthesis are now abundant to enable the preparation of amides as diverse as one can imagine. In this context, developing alternative synthetic approaches which are of enhanced sustainability has become a main issue of present concern in the field of amide synthesis.

As an ideal model of modern organic synthesis, the direct functionalization of inactivated C–H bonds has been proved to be a promising tool to enable atom and step economical synthesis. Inspired by the splendid advances that have taken place in the chemistry of C–H activation, the synthesis of amides has accordingly received significant progress by making use of the coupling between amino group and inert C–H bonds [34–35]. Considering the notable advances that have happened in the research of C–H amidation, it is desirable to provide a review work on this issue. Although different transition metals are known to be capable of catalyzing the C–H amidation reactions, copper is particularly advantageous because of the low cost, low toxicity and broad tolerance of copper catalysts. Therefore, the present review summarizes the advances on the copper-catalyzed C–H activation-based amidation (including related sulfonamidation and imidation) reactions under the categories of C(sp^3^)–H bond amidation, C(sp^2^)–H bond amidation, C(sp)–H bond amidation and cascade reactions initiated by C–H amidation.

## Review

### C(sp^3^)–H bond amidation

#### Intermolecular amidation

**Scheme 1:**
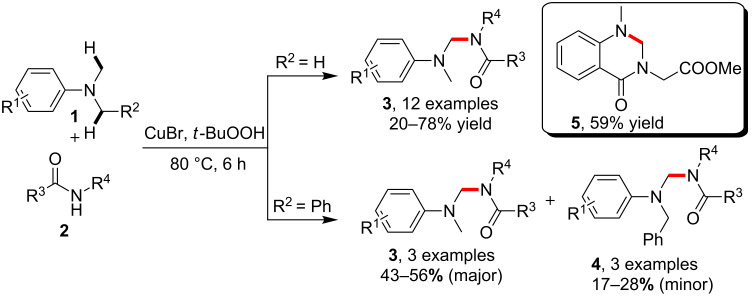
Copper-catalyzed C–H amidation of tertiary amines.

The formation of *N*-alkylamides could be traditionally accessed via nucleophilic *N*-alkylation of amides by using pre-functionalized electrophiles such as alkyl halides, alcohols or amines [36–41]. An alternative tactic which employs raw C–H bond conversion represents a revolutionary step in the synthesis of *N*-alkylamides. In 2007, Fu and co-workers [42] reported the copper-catalyzed, *tert*-butyl hydroperoxide (TBHP)-assisted C–H amidation of tertiary amines **1**. By heating at 80 °C, the C–H bond in dimethylaniline underwent direct amidation to provide products **3** in the presence of amides **2**. On the other hand, the dephenylation transformation via C–C bond cleavage took place as the main route when *N*-phenyl-*N*-methylaniline was employed as the alternative reactant, which led to the production of **3** as the main products, while corresponding products **4** via C–H bond amidation occurred as the minor ones. Notably, this kind of C–H amidation strategy could be utilized for the synthesis of cyclic product **5** via an intramolecular version (Scheme 1).

**Scheme 2:**
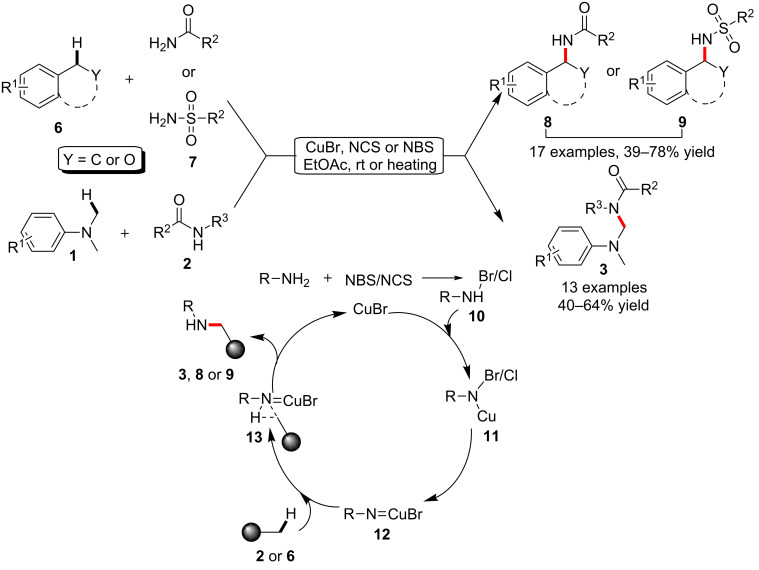
Copper-catalyzed C–H amidation and sulfonamidation of tertiary amines.

On the basis of this successful amidation of the C–H bonds adjacent to a nitrogen atom, the same group later on realized a more generally applicable protocol on the amidation of benzylic C–H bonds and C–H bonds adjacent to oxygen via an *N*-halosuccinimide- (NCS or NBS)-assisted copper-catalyzed process. As shown in Scheme 2, both cyclic and acyclic compounds **6** containing a benzylic C–H bond could be readily converted to *N*-alkylamides **8** or sulfonamides **9** via this much milder catalytic method. In addition, this modified method was also able to allow the amidation of *N,N*-dimethylanilines at room temperature with higher efficiency. One of the key intermediates was proposed to be the *N*-haloamide **10**, which was generated from the incorporation of amides and NBS/NCS. And the subsequent transformation via intermediates **11**, **12** and **13** enabled the final production of the *N*-alkylamides [43].

**Scheme 3:**
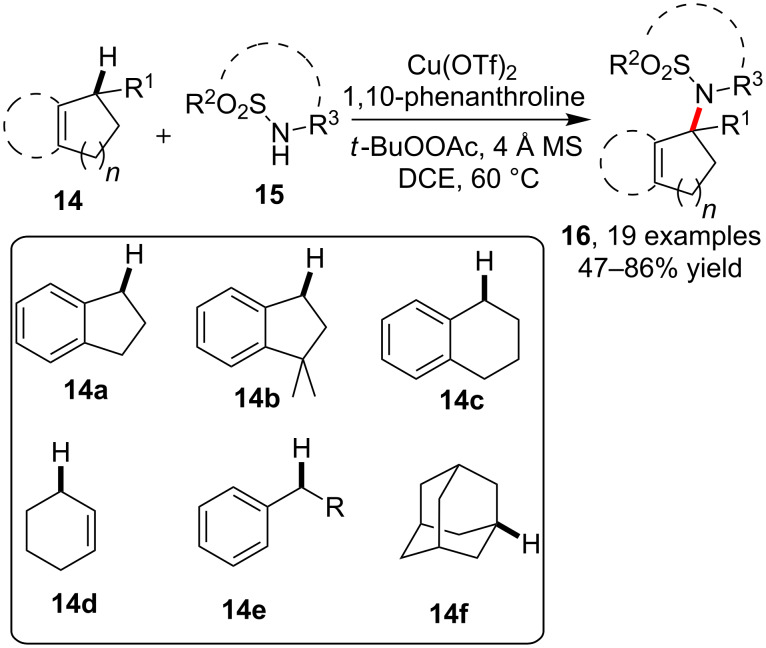
Copper-catalyzed sulfonamidation of allylic C–H bonds.

Also by means of copper catalysis, Powell et al. [44] reported the synthesis of *N*-alkylsulfonamides **16** via the C–H amidation of various sp^3^C–H bonds such as benzylic, allylic and tertiary carbon ones (**14**) in the presence of primary or secondary sulfonamides **15** with the assistance of 1,10-phenanthroline as a ligand (Scheme 3). Notably, the asymmetric version of a similar amidation had been previously achieved by Clark et al. via copper catalysis in the presence of a chiral oxazoline ligand, which allowed the synthesis of enantioenriched products of type **16** [45].

**Scheme 4:**
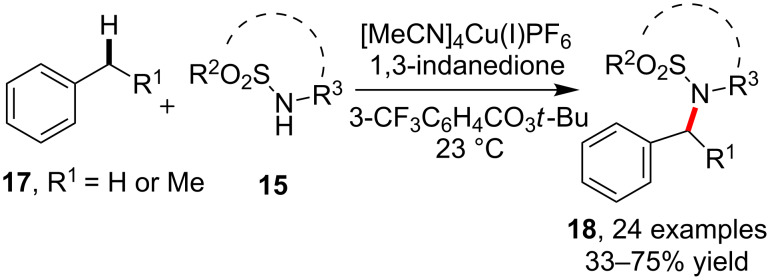
Copper-catalyzed sulfonamidation of benzylic C–H bonds.

In a subsequent study, by modifying the conditions using [MeCN]_4_Cu(I)PF_6_ as copper catalyst and 1,3-indanedione as the ligand, the sulfonamidation of primary benzylic C(sp^3^)–H bonds in toluene were successfully performed at 23 °C in the presence of 3-CF_3_C_6_H_4_CO_3_*t*-Bu, which provides a practical approach to complement the above catalytic version on the sulfonamidation of secondary and tertiary alkyl C–H bonds (Scheme 4) [46].

**Scheme 5:**
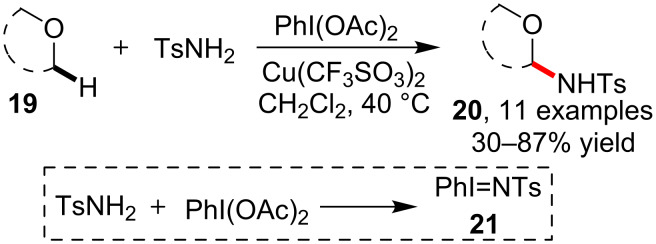
Copper-catalyzed sulfonamidation of C–H bonds adjacent to oxygen.

As an early known tactic with broad application, the nitrene insertion was frequently employed in the sulfonamidation of saturated C–H bonds. However, previously prepared nitrene precursors such as ArI=NTs [47] or chloramine-T [48] were required. To design a facile amidation method using this strategy, Yu and co-worker [49] developed a new method for the synthesis of tosyl-amidated esters **20** via C–H sulfonamidation of cyclic esters **19** under catalysis of copper(II) trifluoromethanesulfonate. The notable advantage of this protocol was that simple tosylamide had been directly used as amide nucleophile. The key point enabling the sulfonamidation transformation was the in situ generation of PhI=NTs (**21**) by employing PhI(OAc)_2_ in the reaction (Scheme 5).

**Scheme 6:**
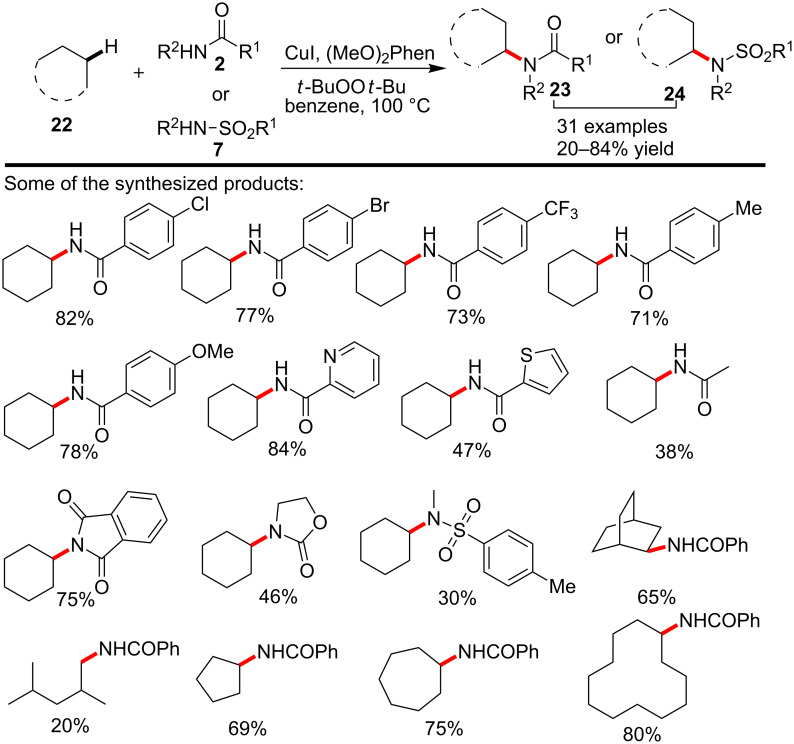
Copper-catalyzed amidation and sulfonamidation of inactivated alkyl C–H bonds.

While most known literature methods in the copper-catalyzed alkane amidation focused on the transformation of either benzylic, allylic C–H bonds or C–H bonds adjacent to nitrogen or oxygen atoms, the amidation of unactivated C–H bonds in simple linear or cyclic alkanes remained as a challenge. Recently, Hartwig and co-workers [50] successfully realized the amidation, sulfonamidation and imidation reactions of purely non-activated cyclic and linear alkanes via the catalysis of copper(I) iodide by employing 4,7-dimethoxyphenanthroline ((OMe)_2_Phen) as the ligand and *t*-BuOO*t*-Bu as an oxidant. Heating the reaction at 100 °C allowed the synthesis of various *N*-alkylamides, sulfonamides and imides with fair to excellent yield, and the results also suggested that the catalytic method tended to selectively enable the transformation of secondary or primary C–H bonds, while the amidation of the tertiary alkyl C–H bond was not favored. More notably, the exploration on the reaction mechanism disclosed that the activation of the alkyl C–H bond was initiated by the *tert*-butoxy radical (Scheme 6).

**Scheme 7:**
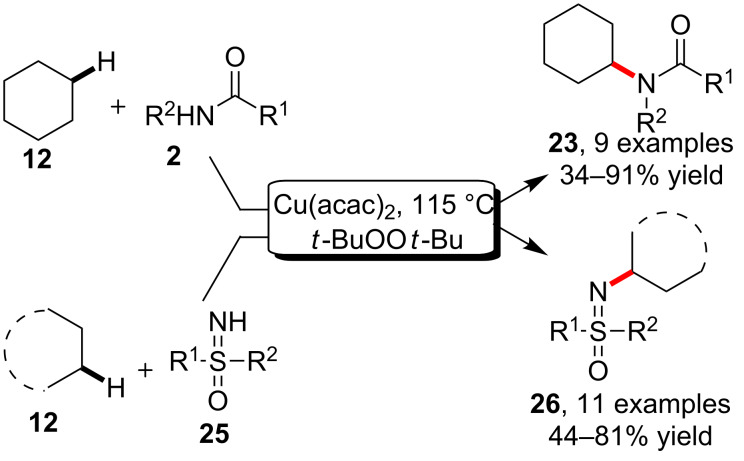
Copper-catalyzed amidation and sulfonamidation of inactivated alkanes.

Inspired by the alkane C–H activation, Yu and Cheng et al. [51] discovered that directly heating amides in cyclohexane in the presence of Cu(acac)_2_ and *t*-BuOO*t*-Bu enabled the C–H amidation for the synthesis of *N*-cyclohexyl amides without using a ligand or an additional solvent. More interestingly, the catalytic method was also efficiently applicable for the *N*-alkylation of sulfoximines for the synthesis of various sulfoximine derivatives (Scheme 7).

#### Intramolecular amidation

**Scheme 8:**
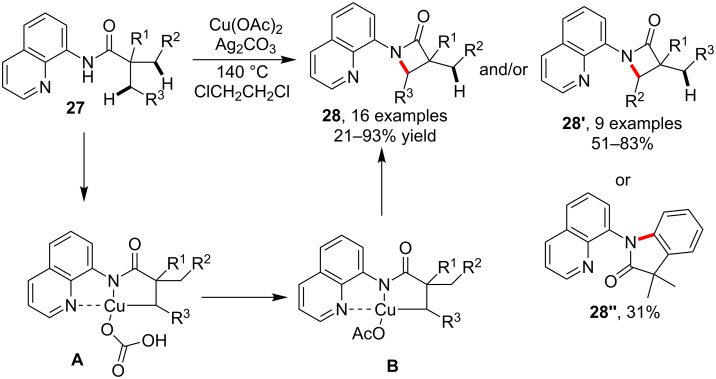
Copper-catalyzed intramolecular C–H amidation for lactam synthesis.

Comparing with the intermolecular amidation, the copper-catalyzed intramolecular version of the sp^3^C–H amidation was much less explored. In 2014, Kuninobu and Kanai et al. [52] reported an unprecedented intramolecular C(sp^3^)–H bond amidation for the synthesis of a lactam via chelating-group-assisted copper catalysis. As outlined in Scheme 8, the *N*-quinolin-8-yl substituted amides **27** could be smoothly transformed into lactams **28** and/or **28'** via C–H amidation by using Cu(OAc)_2_ as catalyst and Ag_2_CO_3_ as a base. In a specific case, the C(sp^2^)–H bond could undertake the amidation to provide indolinone product **28''**. An important factor enabling the C–H bond transformation was the presence of the quinoline auxiliary which acted as a bidentate fragment to incorporate the copper catalyst and facilitate the bond cleavage and formation via intermediates **A** and **B** (Scheme 8).

**Scheme 9:**
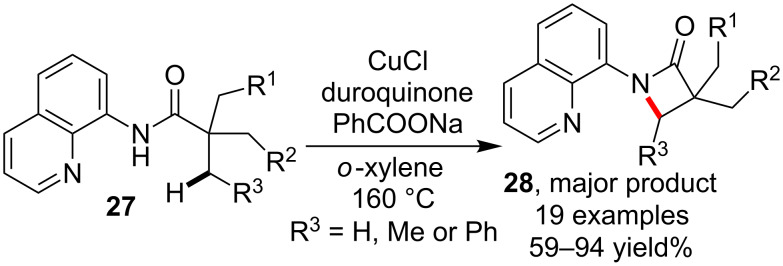
Copper-catalyzed intramolecular C–H amidation for lactam synthesis.

Almost at the same time, Ge et al. [53] reported a similar intramolecular C–H amidation for the synthesis of lactams using CuCl as copper catalyst. When substrates possessing more than one γ-alkyl C–H bond were used, as the case occurred in Kuninobu and Kanai’s work, the primary C–H was preferably transformed over secondary C–H bonds (Scheme 9).

### C(sp^2^)–H bond amidation

**Scheme 10:**
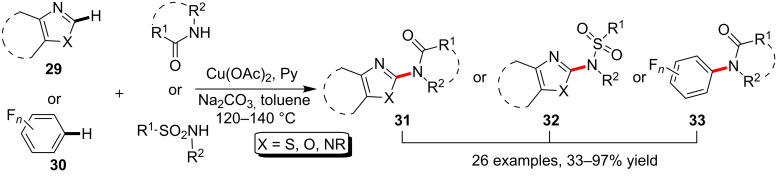
Copper-catalyzed amidation/sulfonamidation of aryl C–H bonds.

The direct transformation of C(sp^2^)–H bonds constitutes an issue of extensive current interest. On the basis of the pioneering work in transition-metal-catalyzed activation of the C(Ar)–H bonds of electron deficient heteroaryls, Schreiber and Wang [54] attempted and achieved the Cu(OAc)_2_-catalyzed C–H amidation/sulfonamidation of azoles **29** and polyfluorinated arenes **30** under the assistance of pyridine (Py) as ligand and base. Corresponding products **31**, **32** and **33** were readily acquired with fair to excellent yield depending on the properties of both aryl substrate and amide nucleophile (Scheme 10). It is worth to mention that Pan and co-workers recently disclosed *N*-fluorobenzene sulfonimides to be employed as the source of sulfonamide to enable the synthesis of *N*-heteroaryl sulfonimides via copper-catalyzed aryl C–H sulfonimidation [55].

**Scheme 11:**
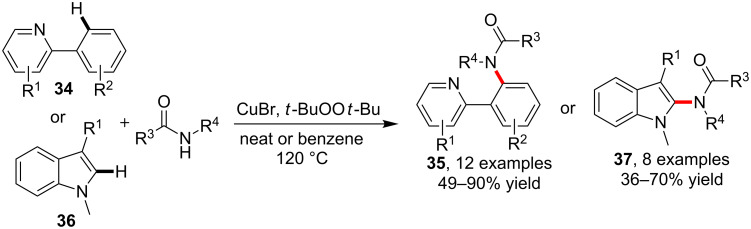
C–H amidation of pyridinylbenzenes and indoles.

In contrast to the direct transformation of the weakly acidic C–H bonds in these electron deficient arenens/heteroarenes, the amidation of normal aryl C–H bonds usually relied on the presence of a directing group (DG). In 2010, Li et al. [56] reported the *o*-amidation of 2-arylpyridines **34** via the catalysis with CuBr and oxidation with *tert*-butyl peroxide. Besides the application of *N*-substituted and unsubstituted amides in the synthesis of **35**, the sulfonamidation using TsNH_2_ was also successfully performed. In addition, this copper-catalyzed amidation protocol was also found to be applicable for the synthesis of 2-amino-1-methylindoles **37** via C–H amidation of indoles **36** by employing benzene as the medium (Scheme 11).

**Scheme 12:**
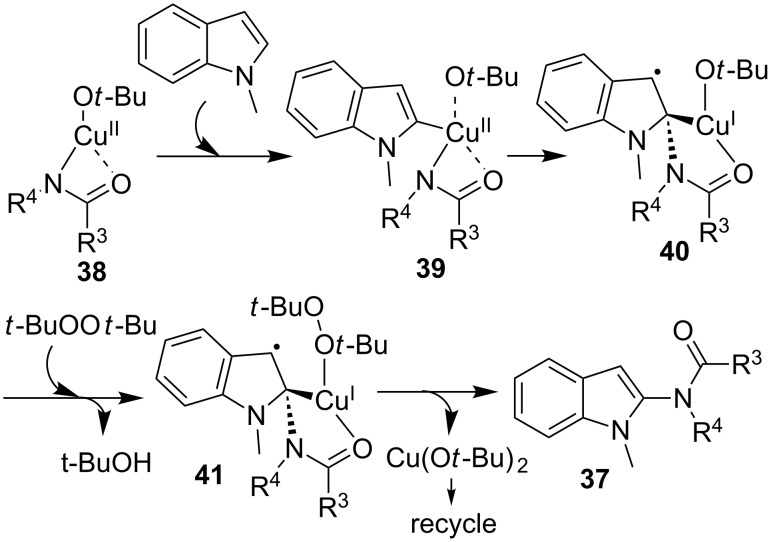
Mechanism of the Cu-catalyzed C2-amidation of indoles.

While the authors proposed that the mechanism in the selective C-2 amidation of *N*-methylindoles resulted from a classical oxidative addition/reductive elimination Cu(III)/Cu(I) process, Himo and co-workers [57] provided a different Cu(II)/Cu(I) mechanism to explain the selectivity of this C-2 amidation transformation based on the study with DFT calculation. As outlined in Scheme 12, the calculation results suggested that the C-2 amidation of indole was possibly initiated by the Cu(II)-based bidentated intermediate **38**, which proceeded via a series of different intermediate states **39**–**41** to provide products **37** in the presence of *tert*-butyl peroxide.

**Scheme 13:**
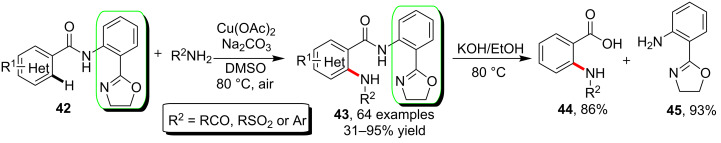
Copper-catalyzed, 2-phenyl oxazole-assisted C–H amidation of benzamides.

By means of the assistance of molecular oxygen, Nicholas and John [58] devised the copper-catalyzed 2-amidation and sulfonamidation of 2-arylpyridines via C–H activation. Besides the peroxide-free advantage, the C–H amination using aniline was found applicable to allow the synthesis of biarylamine. More recently, based on the DG strategy, the Yu group [59] designed the *o*-amidation of arylamides with copper catalysis under aerobic conditions. Upon systematic screening, the 2-phenyloxazole structure was found as an excellent DG to enable the *ortho*-C–H functionalization of amide **42** to provide products **43**. Under the assistance of this novel DG, this catalytic method exhibited exceptionally broad tolerance to the C–H functionalization with nitrogen nucleophiles, including amides, sulfonamides and primary arylamines. In addition, the oxazole-based DG could be easily deprotected to provide the corresponding benzoic acid **44** by heating in EtOH in the presence of KOH (Scheme 13).

**Scheme 14:**
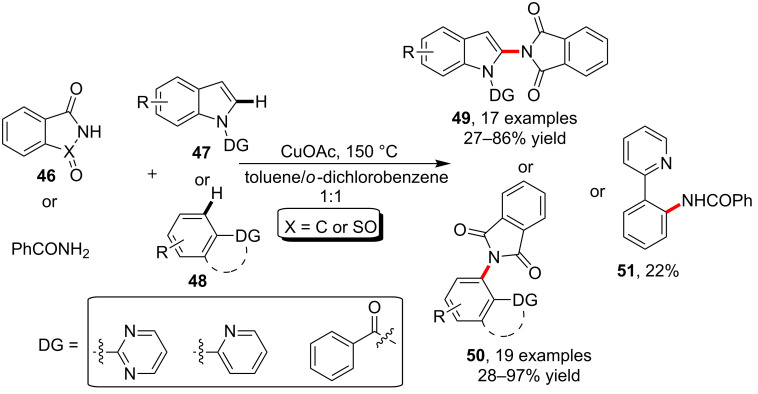
DG-assisted amidation/imidation of indole and benzene C–H bonds.

As another easily available *N*-containing aromatic heterocycle, the pyrimidine ring was disclosed as useful DG in copper-catalyzed C–H activation. As reported by Shen and co-workers [60], the C–H bond in indoles **47** and benzenes **48** could be effectively activated with copper in the presence of DGs such as pyrimidin-2-yl, pyridine-2-yl or benzoyl to provide products **49** or **50** by incorporating phthalimide/saccharin **46**. Under the standard conditions, however, the reaction of benzamide with 2-phenylpyridine provided product **51** with low yield (Scheme 14).

**Scheme 15:**
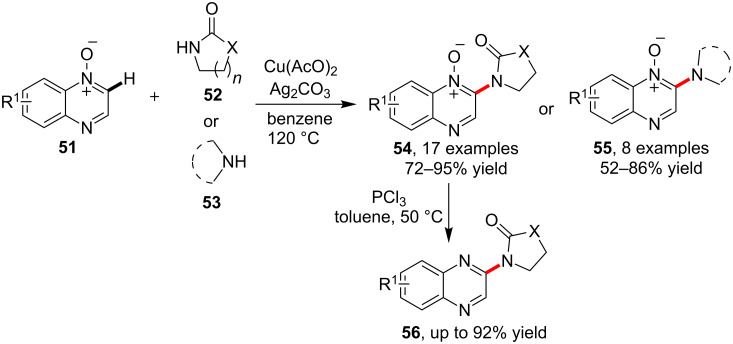
Copper-catalyzed C–H amination/amidation of quinoline *N*-oxides.

As a special aromatic system, quinoline *N*-oxides were well investigated in their reactivity for metal-catalyzed C–H activation. Based on the known results of quinoline *N*-oxide C–H alkenylation [61], arylation [62] and alkylation [63] etc, Li and co-workers [64] investigated and established the C–H amidation of quinoline *N*-oxides **52** via copper catalysis. According their results, quinoline *N*-oxides **51** underwent C–H amidation with lactams **52** to yield 2-aminoquinoline *N*-oxides **54** with generally excellent yield. Notably, the catalytic system also allowed a C–H bond amination by using secondary amines **53** for the synthesis of 2-aminoquinoline *N*-oxides **55**. What’s more, the *N*-oxides could be efficiently reduced to give the corresponding quinoline derivatives **56** by simply treating **54** with PCl_3_ (Scheme 15).

**Scheme 16:**
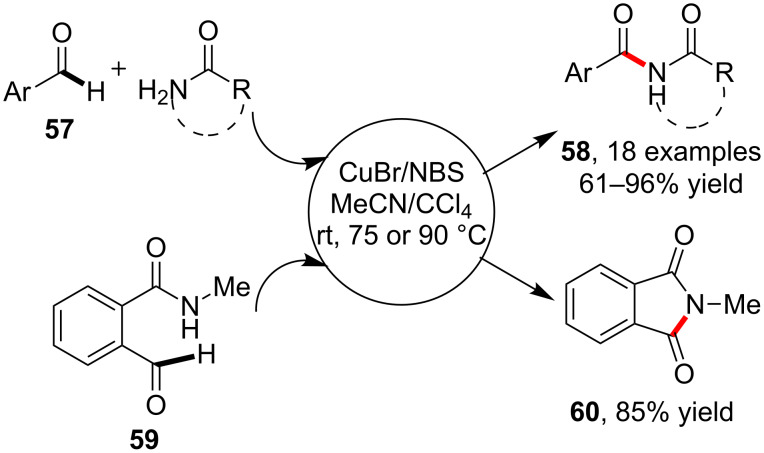
Copper-catalyzed aldehyde formyl C–H amidation.

As another kind of conventional unsaturated hydrocarbons, the alkenes had been found to show similar reactivity with arenes in many cross coupling reactions. However, in the C–H amidation chemistry, the copper-catalyzed reactions of alkenes were rarely available. An interesting point was that the C(sp^2^)–H bond at the C=O double bond had been known to possess the reactivity toward amidation via copper catalysis. In 2008, Fu and co-workers [65] reported the carbonyl C–H amidation of aryl aldehydes via the catalysis of CuBr and NBS. By simply stirring at rt, 75 or 90 °C, a variety of primary amides and lactams incorporated aldehydes **57** to yield imides **58** with moderate to excellent yield. In addition, the method also displayed sound application in the synthesis of cyclic imides such as *N*-methylphthalimide (**60**) via the intramolecular amidation of *N*-methyl-*o*-formylbenzamide (**59**) (Scheme 16).

**Scheme 17:**
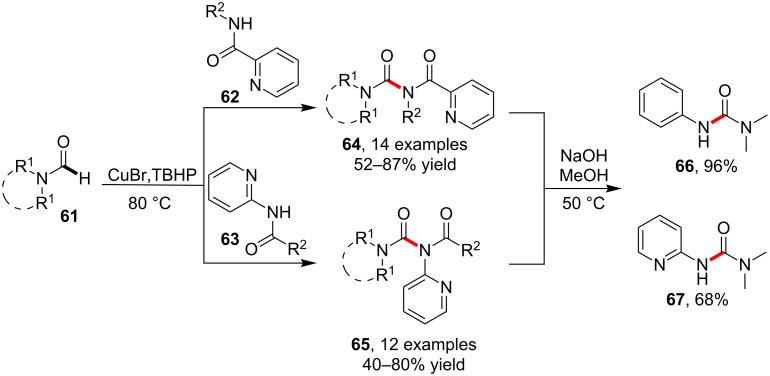
Copper-catalyzed formamide C–H amidation.

More recently, Lan et al. [66] realized the C–H amidation of *N,N*-dialkylformamides **61** using pyridine-2-yl-functionalized amides **62** or **63**. Under catalytic conditions consist of CuBr and TBHP, imides **64** and **65** were afforded, respectively. The imide products **64** and **65** could both be efficiently hydrolyzed to provide ureas **66** and **67**. The presence of the pyridine ring in substrates **62** and **63** was crucial for the conversion of the inert carbonyl C–H bond in **61** by chelating the copper catalyst (Scheme 17).

**Scheme 18:**
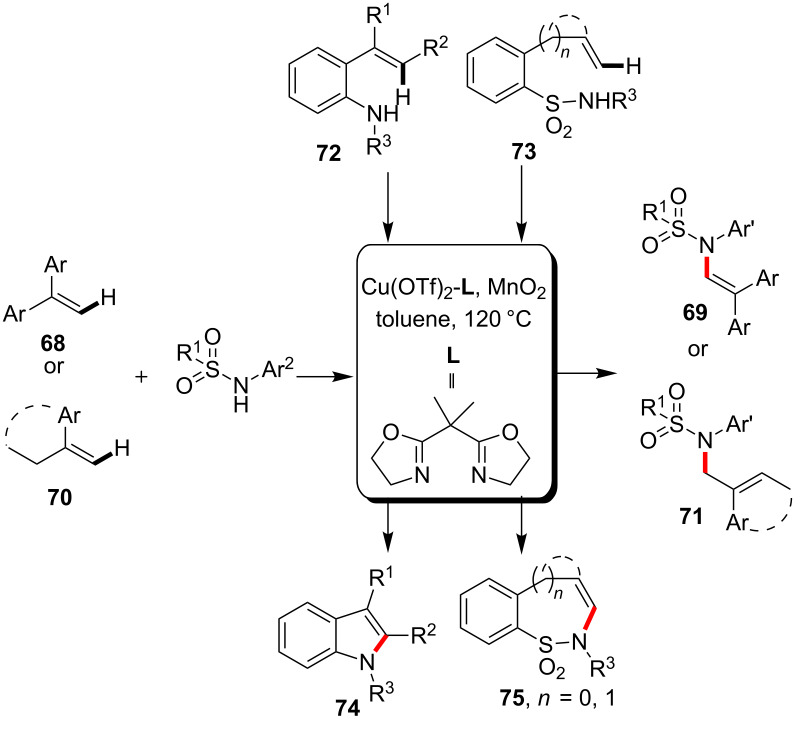
Copper-catalyzed sulfonamidation of vinyl C–H bonds.

The C–H bond sulfonamidation of alkene substrates were systematically investigated by Chemler and co-workers. For example, they [67] developed the copper-catalyzed protocol for the sulfonamidation reaction of alkenes **68** via direct C–H activation to provide *N*-vinylsufonamides **69** in the presence of an oxazoline ligand. On the other hand, the reaction of allylic substrates **70** gave *N*-allylsulfonamides **71**. The catalytic approach was also well tolerable to the intramolecular version for the synthesis of indoles **74** and cyclic sulfonamides **75** by using **72** and **73** as starting materials, respectively (Scheme 18). The intramolecular version of the reaction in the synthesis of indoles was later achieved by mean of ligand-free condition via the co-catalysis of Cu(eh)_2_ (copper(II) 2-ethylhexanoate) and TEMPO under oxygen atmosphere [68].

### C(sp)–H bond amidation

**Scheme 19:**
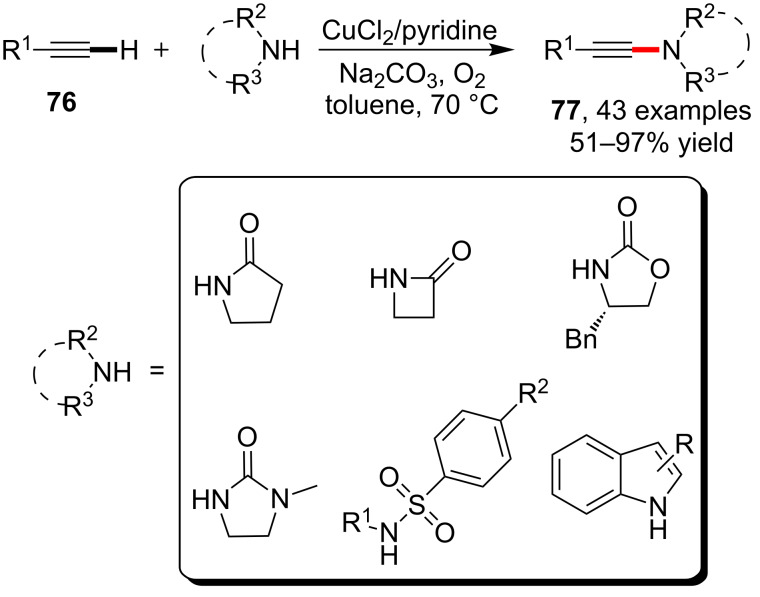
CuCl_2_-catalyzed amidation/sulfonamidation of alkynyl C–H bonds.

The C(sp)–H bond in terminal alkynes is more acidic than equivalent alkane and alkene C–H bonds, and the alkynylation reactions by making use of direct transformations on the alkyne C–H bonds such as Sonograshira, Glaser couplings were extensively studied and utilized [69–72]. Under the inspiration of these well-known reaction models, the amidation reaction based on the activation of alkyne C–H bonds were also implemented. In 2008, Stahl et al. [73] reported the first copper-catalyzed alkyne amidation via the oxidation with molecular oxygen. The synthetic protocol exhibited excellent tolerance to the C–H functionalization by reacting not only with lactams, but also with cyclic imides, carbamates, sulfonamides and indoles. On the other hand, the successful amidation using different alkynes, including aryl-, alkyl- and silyl-functionalized alkynes proved the broad scope of application of this method (Scheme 19).

**Scheme 20:**
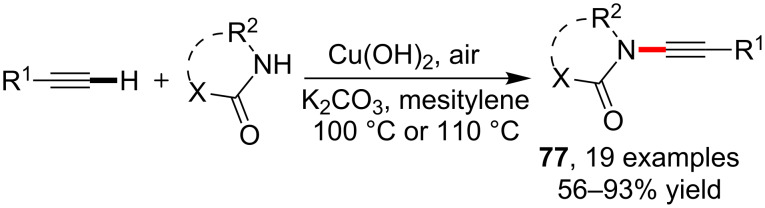
Cu(OH)_2_-catalyzed amidation/sulfonamidation of alkynyl C–H bonds.

Following the design of this method, a heterogeneous catalytic protocol was later developed by Mizuno et al. [74] for the amidation of terminal alkynes using lactam, sulfonamide or cyclic carbamates. The application of Cu(OH)_2_ as heterogeneous catalyst allowed the synthesis of ynamides **77** with moderate to excellent yield under air (Scheme 20). A latest work on this area from Truong et al. [75] showed that the heterogeneous and recyclable Cu_2_(BDC)_2_(BPY) catalyst (BDC = benzene- 1,4-dicarboxylate; BPY = 4,4′-bipyridine) could catalyze this kind of amidation reaction with excellent selectivity to provide ynamides. Another point was that the C–H bond could also get aminated by using secondary amines such as diphenylamine.

### C–H bond amidation in cascade reactions

**Scheme 21:**
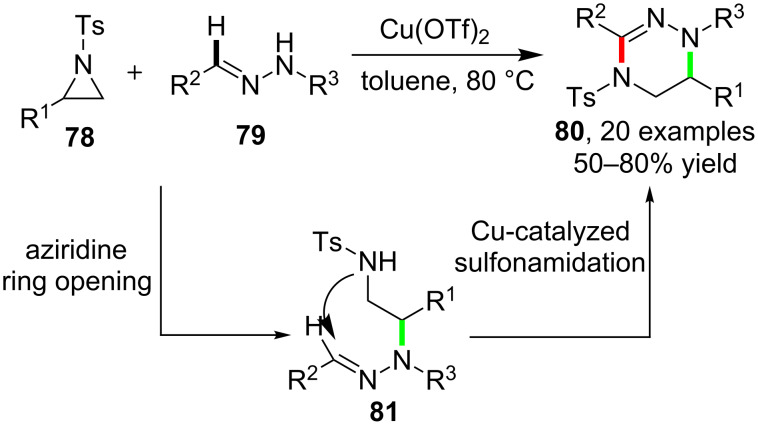
Sulfonamidation-based cascade reaction for the synthesis of tetrahydrotriazines.

The success of these different kinds of C–H amidation reactions, as aforementioned, enabled the facile synthesis of diverse secondary or tertiary amides by installing a new chemical motif to the nitrogen atom. On the other hand, designing cascade reactions by employing the C–H amidation constituted another issue of extensive interest since these reactions enabled the construction of more complex and diverse products via the formation of multiple chemical bonds in one step operation. Early in 2009, Lin and Wang et al. [76] reported the cascade reactions between *N*-tosylaziridines **78** and hydrazones **79** which led to the synthesis of tetrahydrotriazines **80**. The cascade aziridine ring opening and copper-catalyzed intramolecular C–H sulfonamidation via intermediate **81** characterizes the whole reaction process (Scheme 21).

**Scheme 22:**
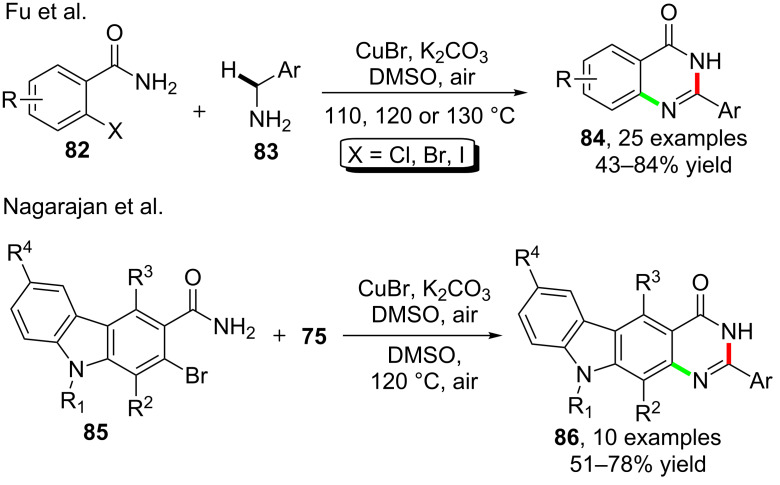
Copper-catalyzed cascade reaction for the synthesis of quinazolinones.

During their efforts in developing cascade reactions for the synthesis of heterocycles, Fu and co-workers [77] established a method for the synthesis of quinazolinones **84** through the reactions between *o*-halobenzamides **82** and benzylamines **83**. The formation of the target products were realized via a tandem Ullmann-type C–N coupling of the Ar–X bond and the amino group in **83** as well as the intramolecular amidation which was believed to assist the oxidative formation of the imine C=N bond (Scheme 22). By making use of this cascade synthetic method, Nagarajan et al. [78] finished the synthesis of various polycyclic structured quinazolinones **86** via corresponding starting materials **85** which were synthesized before by stepwise preparation (Scheme 22).

**Scheme 23:**
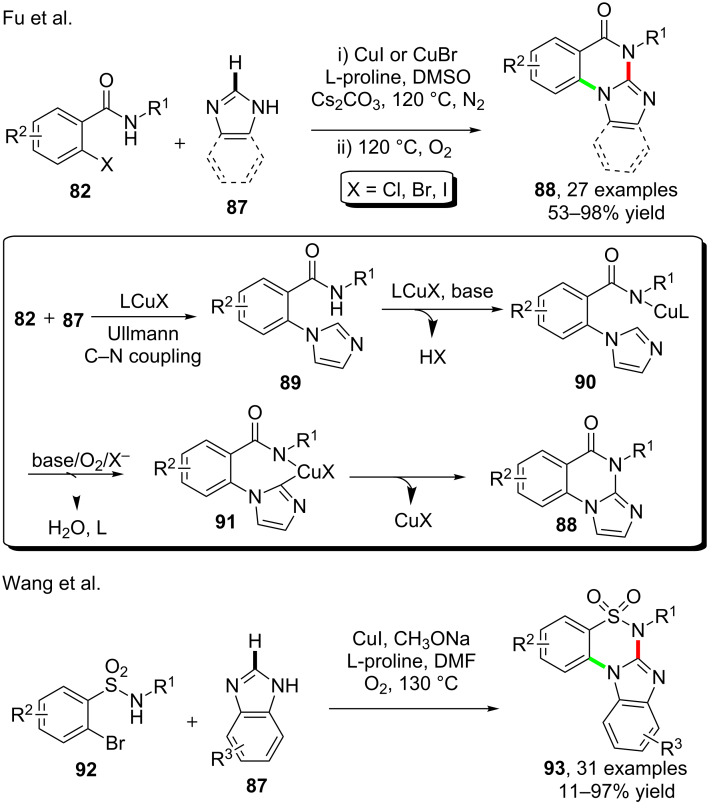
Copper-catalyzed cascade reactions for the synthesis of fused quinazolinones.

Based on a similar strategy of combining an Ullmann C–N bond formation and C–H amidation, Fu and Xu [79] also achieved the cascade reactions of o-halobenzamides **82** and (benzo)imidazoles **87** for the one-pot synthesis of (benzo)imidazoquinazolinones **88** under the catalysis of CuI and assistance of L-proline. A subsequent oxidation using molecular oxygen was required for the final formation of products. According to the results, the mechanism of the cascade reactions was proposed as shown in Scheme 23 wherein the intermediate **89** generated by Ullmann C–N coupling reaction, the bidentate copper complex **90** and **91** were assumed as the key stages of the cascade reaction. Recently, cascade reactions using analogous *o*-halobenzenesulfonamide **92** were disclosed by Wang et al. [80]. The synthesis using **92** and benzimidazoles **87** provided benzimidazole-fused cyclic sulfonamides **93**. The reaction allowed the synthesis of various products with fair to high yields with the assistance of L-proline as ligand. The expected conversion took place also in the absence of a ligand, but with evidently lower yield than the equivalent reaction with ligand (Scheme 23).

**Scheme 24:**
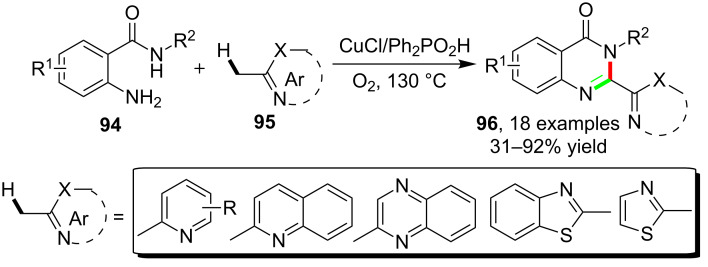
Copper-catalyzed synthesis of quinazolinones via methyl C–H bond amidation.

Based on a novel cascade reaction, the synthesis of quinazolinones was realized by employing *o*-aminobenzamides and methylated electron-deficient heterocycles. As reported by Han et al, the catalysis of CuCl enabled the cascade reactions between *o*-aminobenzamides **94** and 2-methylheteroaryls **95** and led to the synthesis of quinazolinones **96** via the formation of C=N and C–N bonds in the presence of Ph_2_PO_2_H and oxygen. For the methylated component **95**, the application scope of the synthesis focused on the electron deficient reactants such as 2-methylpyridine, 2-methylquinoline, 2-methylquinoxalines, 2-methylthiazole and 2-methylbenzothiazole, other methylated heterocycle such as 3-methylpyridine was not tolerated (Scheme 24) [81].

**Scheme 25:**
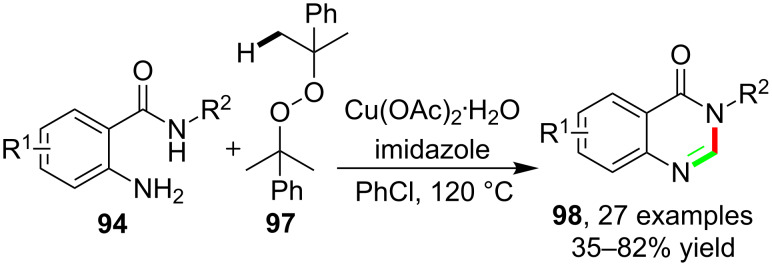
Dicumyl peroxide-based cascade synthesis of quinazolinones.

Similar quinaxolinone products were also successfully synthesized by the incorporation of *o*-aminobenzamides and a novel peroxide-based methyl donor. Wang and co-workers [82] found that the reactions of **94** with dicumyl peroxide **97** could provide 2-unsubstituted quinazolinones **98** with broad diversity. In these reactions, the dicumyl peroxide acted as the methyl donor to provide a methyl radical as the precursor of the methyl cation which was crucial for the subsequent annulation. The radical process of the reaction was also supported by the EPR experiment (Scheme 25).

**Scheme 26:**
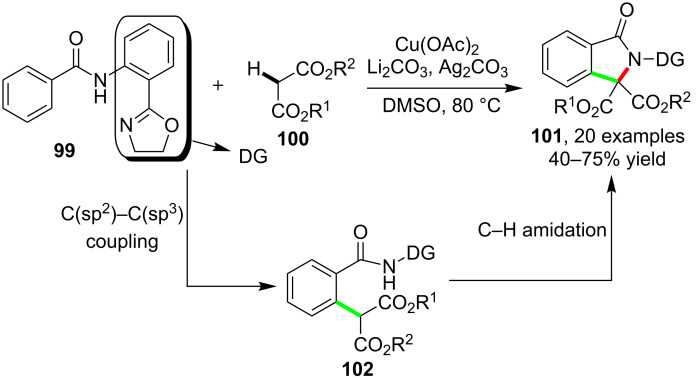
Copper-catalyzed cascade reactions for the synthesis of indolinones.

While most of the known C–H amidation-based cascade reactions afforded six-membered or related ring-fused products, the formation of other ring sized scaffolds such as five-membered structure were much less. An interesting cascade reaction between DG-functionalized benzamides **99** and malonates **100**, which enabled the synthesis of indolinones **101** via selective C–C and C–N bond construction, was reported by Dai and Yu et al. [83]. As outlined in Scheme 26, the presence of the oxazole-based DG was found to be the main factor in tuning the selective formation of **101**. Related control experiments suggested that the formation of intermediate **102** acquired from the copper-catalyzed C(sp^2^)–C(sp^3^) bond formation was the key transformation of the cascade process.

## Conclusion

On the basis of the magnificent advances taking place in the chemistry of the C–H activation, the synthesis and elaboration of amides has won new opportunity via a more economical and simple process. With the copper-catalyzed amidation of C(sp^3^)–H, C(sp^2^)–H, C(sp)–H bonds, a large variety of different products have been successfully synthesized via either single bond formation or cascade construction of more than one bond. These successful examples convincingly demonstrated the application potential of the C–H amidation in organic synthesis. On the other hand, it should also be noted that challenges still remain in presently known results of C–H amidations. For example, the copper-catalyzed amidation of olefinic C(sp^2^)–H bonds is still hardly available, and the heating to high temperatures for most of the C–H amidation-based transformation is another point demanding urgent improvement. The significance of the C–H amidation reactions, together with the unsolved problems in the known investigations, discloses the huge chemical space remained in the research field, and much more interesting results are expected in future from the chemistry related to the C–H amidation.
